# A *Drosophila*-inspired intelligent olfactory biomimetic sensing system for gas recognition in complex environments

**DOI:** 10.1038/s41378-024-00752-y

**Published:** 2024-10-28

**Authors:** Xiawei Yue, Jiachuang Wang, Heng Yang, Zening Li, Fangyu Zhao, Wenyuan Liu, Pingping Zhang, Hong Chen, Hanjun Jiang, Nan Qin, Tiger H. Tao

**Affiliations:** 1 State Key Laboratory of Transducer Technology, Shanghai Institute of Microsystem and Information Technology, Chinese Academy of Sciences, Shanghai, 200050 China; 2 School of Graduate Study, University of Chinese Academy of Sciences, Beijing, 100049 China; 3 Suzhou Huiwen Nanotechnology Co. Ltd., Jiangsu, 215004 China; 4 School of Integrated Circuits, Tsinghua University, Beijing, 100084 China; 5 Center of Materials Science and Optoelectronics Engineering, University of Chinese Academy of Sciences, Beijing, 100049 China; 6 2020 X-Lab, Shanghai Institute of Microsystem and Information Technology, Chinese Academy of Sciences, Shanghai, 200050 China; 7 Center for Excellence in Brain Science and Intelligence Technology, Chinese Academy of Sciences, Shanghai, 200031 China; 8 Neuroxess Co. Ltd. (Jiangxi), Nanchang, Jiangxi 330029 China; 9 Guangdong Institute of Intelligence Science and Technology, Hengqin, Zhuhai, Guangdong 519031 China; 10 Tianqiao and Chrissy Chen Institute for Translational Research, Shanghai, China

**Keywords:** Electrical and electronic engineering, Materials science

## Abstract

The olfactory sensory system of *Drosophila* has several advantages, including low power consumption, high rapidity and high accuracy. Here, we present a biomimetic intelligent olfactory sensing system based on the integration of an 18-channel microelectromechanical system (MEMS) sensor array (16 gas sensors, 1 humidity sensor and 1 temperature sensor), a complementary metal‒oxide‒semiconductor (CMOS) circuit and an olfactory lightweight machine-learning algorithm inspired by *Drosophila*. This system is an artificial version of the biological olfactory perception system with the capabilities of environmental sensing, multi-signal processing, and odor recognition. The olfactory data are processed and reconstructed by the combination of a shallow neural network and a residual neural network, with the aim to determine the noxious gas information in challenging environments such as high humidity scenarios and partially damaged sensor units. As a result, our electronic olfactory sensing system is capable of achieving comprehensive gas recognition by qualitatively identifying 7 types of gases with an accuracy of 98.5%, reducing the number of parameters and the difficulty of calculation, and quantitatively predicting each gas of 3–5 concentration gradients with an accuracy of 93.2%; thus, these results show superiority of our system in supporting alarm systems in emergency rescue scenarios.

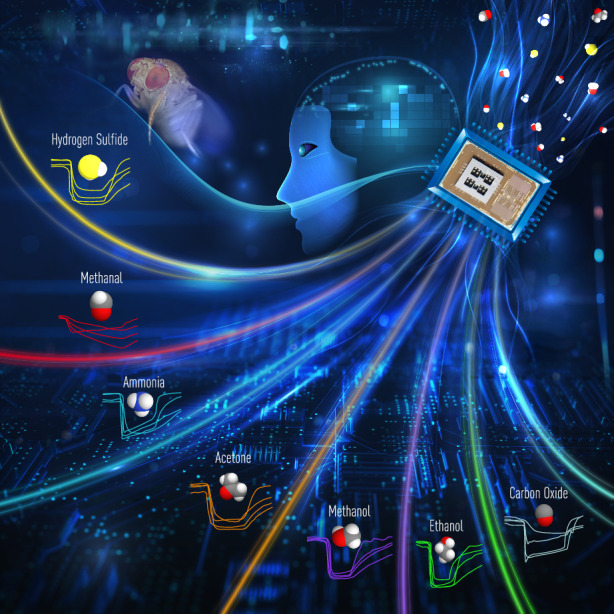

## Introduction

The bionic gas sensing system is an analytical tool for gas detection that mimics biological olfactory capabilities and generates distinctive fingerprint data for various gases^1^. Since this system was first developed by Wilkens and Hatman^2^, it has undergone significant advancements^3,4^ in diverse industrial applications, including indoor air quality monitoring^5^, medical care^6–8^, hazardous gas detection, environmental quality monitoring^9^ and food quality control^10^. To detect various odors, a variety of gas sensors have been examined and developed. Currently, semiconductor gas sensors are widely used due to their high sensitivity, fast response, and low cost^11,12^. Furthermore, due to their relatively low manufacturing cost and compact size, they are compatible with microelectronic processes^13,14^. This provides an economical and efficient solution for gas detection. These sensors typically consist of two main parts: a microheater^15^ and a gas-sensitive material^16^. The primary function of the microheater is to provide the gas-sensitive material with an appropriate operating temperature to optimize its detection performance^17,18^. Common metal oxide semiconductor (MOS) materials include SnO_2_, ZnO, WO_3_, and TiO_2_.

Currently, the significance of the research and development of gas sensing technology is steadily increasing^19–24^. However, the integration of sensor arrays, circuits, and algorithms increases the size and power consumption^3,25,26^; thus, coordination between modules is essential for achieving system-level performance improvement^26–29^. These challenges predominantly stem from the complexities associated with the integration process and the limited fault tolerance inherent in current designs^30^. Afridi^31^ designed a gas sensing system consisting of four microheater gas sensors and achieved monolithic integration. In contrast, the multichip approach improves the flexibility of the system by separately designing sensors and circuits. Therefore, considering practical application flexibility and cost, many researchers opt to design a gas sensor and a custom circuit chip via a multichip approach^32^.

Combining multiple sensors with multivariate data processing methods is one of the most promising approaches for enhancing the selectivity of gas sensors and achieving high-performance intelligent olfactory perception systems^33,34^. Artificial neural networks (ANNs), inspired by the animal brain, have also gained significant popularity in recent years^35,36^, mainly due to substantial improvements in computing power and the advantages of big data^37^. Various types of ANNs, such as multilayer perceptrons and learning vector quantization, have been used for data classification. However, to improve the accuracy of gas recognition and classification, many of the algorithms require a substantial amount of training data to optimize their parameters and model structure. Since the gas testing process is relatively time-consuming, identifying and classifying gas information efficiently and accurately using limited gas data presents a new challenge for algorithms. *Drosophila* are considered one of the fastest-reacting organisms in nature. The olfactory circuitry generates a specific label for each type of gas, which is crucial for learning and responding to different odors. Based on the *Drosophila* olfactory mechanism, the locality-sensitive hashing algorithm is utilized for gas type identification and odor concentration prediction.

In this study, we report an 18-channel sensor array integrated with CMOS readout circuits and a *Drosophila*-inspired algorithm to construct a biomimetic electronic olfactory sensing system that mirrors the functionality of biological olfactory cells, the nervous system, and the brain. The odor information is gathered and then processed by reproducing the biological procedures of *Drosophila*. This system enables the accurate detection of the environmental temperature, humidity, type and concentration of noxious gas. We aim to use an integrated electronic olfactory sensing system to achieve noxious gas identification and support applications in health care, environment monitoring, and intelligent alarm systems in emergency rescue as a proof-of-concept.

## Results

### Biomimetic design of the electronic olfactory sensing system

**Fig. 1:**
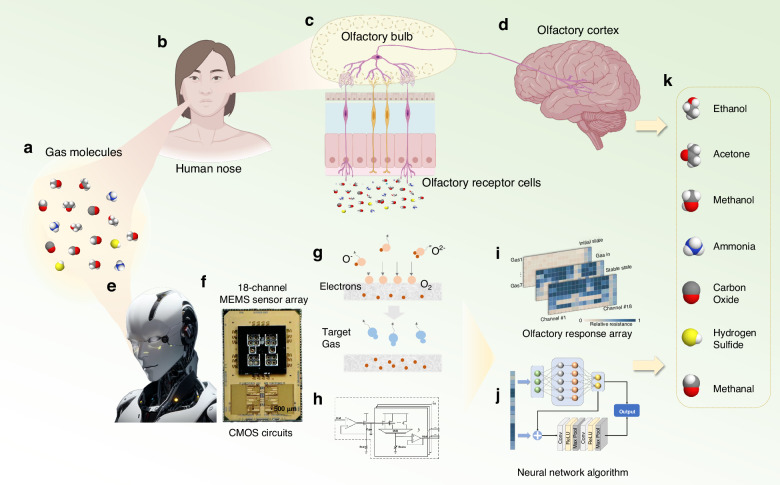
Biomimetic intelligent electronic nose sensing system. **a**–**d** Biological olfactory perception pathway. **e** Biomimetic intelligent olfactory sensing system. **f** Integrated sensing system consisting of the MEMS sensor arrays and CMOS processing circuit chips. **g** Gas sensing mechanisms of metal oxide semiconductor gas-sensitive materials. **h** CMOS readout circuit design. **i** Olfactory response arrays for different odor molecules. **j** The algorithm design for the identification of gas types and prediction of odor concentrations. **k** Gas molecules used in this study

Biology involves a remarkable olfactory system capable of detecting and distinguishing millions of odors^38^. This capability stems from a unique encoding-decoding-combination strategy employed by olfactory receptors and neural networks within the brain^39^. Odor molecules from the environment dissolve in the mucus on the olfactory epithelium (Fig. 1a, b) and then bind to specific olfactory receptors on olfactory neurons. Each receptor is sensitive to particular gas molecules, thereby initiating an electrical response. The electrical signals generated are further transmitted through the olfactory nerves to the olfactory bulb^40^ for the initial processing of olfactory information (Fig. 1c). Subsequently, the processed signals are relayed to the olfactory cortex (Fig. 1d) for comprehensive processing and discrimination of odors.

The general layout of the biological olfactory organs was retained in our design. Figure 1f shows that the biomimetic intelligent olfactory sensing system is composed of an 18-channel MEMS sensor array (gas detection) and CMOS acquisition circuits (information processing). A MEMS multisensor array (16 gas sensors, 1 temperature sensor and 1 humidity sensor) is fabricated based on a microhotplate^41–43^ with various MOS gas-sensitive nanomaterials (Fig. 1g). The CMOS acquisition circuits simulate the human olfactory mechanism to gather and process odor information (Fig. 1h). In the end, by emulating the olfactory receptors of *Drosophila*, each odor is assigned a distinct label. Subsequently, by employing a sparse, binary random matrix to increase data dimensionality (Fig. 1i), we aim to utilize a greater number of neurons to represent objects within the database. Next, winner-takes-all mechanisms is employed for dimensionality reduction, facilitating the rapid identification and recognition of unknown gases (Fig. 1j).

### Fabrication and functionality of the 18-channel MEMS sensor array

**Fig. 2:**
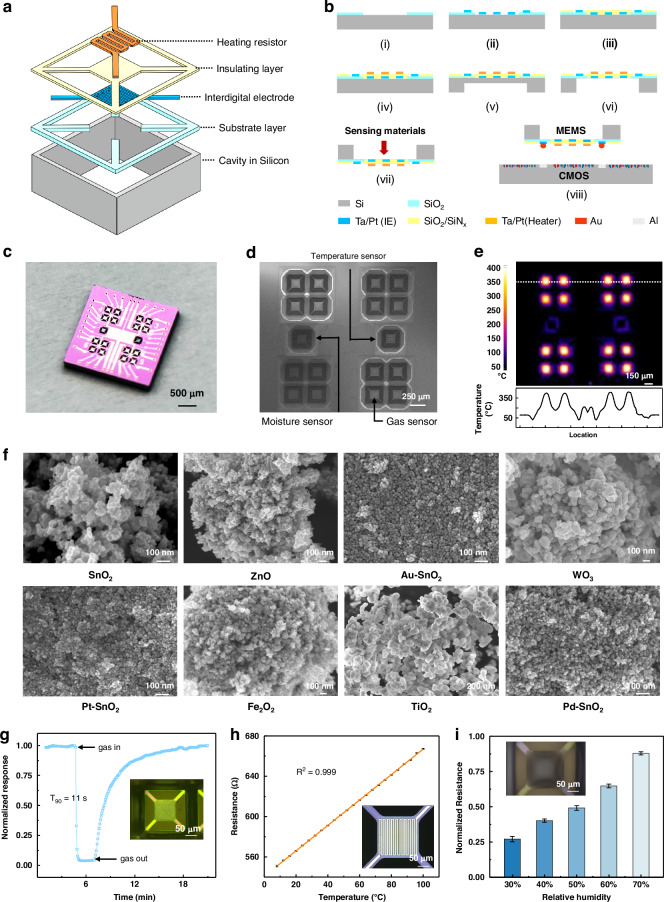
Structure and characterization of the MEMS gas sensor array. **a** Design of a single MEMS gas sensor comprises, from top to bottom, a heating resistance wire, an isolation layer, interdigital electrodes, an insulated layer, and a deep cavity within the silicon substrate. **b** Corresponding fabrication process of the microsensor array in cross-sectional views. (i) Deposition of SiO_2_ via dry etching. (ii) Patterning 30/300 nm Ta/Pt interdigital electrode through sputtering and a lift-off process. (iii) Deposition of SiO_2_ and SiNx through plasma enhanced chemical vapor deposition (PECVD); then electrode pads of interdigital electrode are expose by dry etching. (iv) Patterning Ta/Pt heating resistor through sputtering and lift-off process. (v) Deep etch for silicon from the back of wafer. (vi) Release of the suspended membrane in TMAH solution. (vii) Au bumping with a diameter of ~50 μm and inkjet printing with metal oxide sensing materials. (viii) MEMS gas sensor array connected to a CMOS device by flip-chip bonding. **c** Optical image of the fabricated MEMS gas sensor array. **d** Backside view of the 16 independent gas sensing channels, 1 temperature sensor, and 1 humidity sensor. **e** Infrared thermal image under a total current of 0.32 A with the temperature distribution along the white dotted line. **f** Scanning electron microscopy images of the metal oxide-sensing nanomaterials. **g** Single-channel gas sensor for 10 ppm acetone. **h** Platinum resistance against temperature. **i** Test results of the humidity sensor

In the 18-channel MEMS sensor array, each gas sensor consists of a sophisticated five-layered structure, with a heating resistor, an insulated layer, an interdigital electrode (IE), a substrate layer, and a silicon cavity (Fig. 2a). The fabrication process of the device is depicted in Fig. 2b and the Experimental section. A platinum heater, which is strategically incorporated for high-temperature operation, plays a vital role in optimizing the sensitivity of a gas sensor unit. Following dry etching and anisotropic wet etching, the platinum interdigital electrode is exposed, and suspension beams are constructed to curtail heat loss and foster energy conservation. However, the potential issues stemming from thermal expansion-induced deformation of the supporting film need to be addressed because they can intensify the stress at the joint of the supporting suspension beam and the substrate frame. To increase the mechanical strength of the microheater, we implemented an enhanced angle compensation mechanism at the connection (Figure S1a). This innovative approach ensures that the angle of the connection is cambered, effectively mitigating stress induced by deformation and improving the mechanical stability. Overall, these design and fabrication processes aim to create MEMS sensor arrays with high sensitivity, high stability, and diverse working temperatures for different sensing materials. The use of platinum heaters and careful engineering of structural components have led to efforts to optimize performance and reliability.

Our MEMS sensor array (Fig. 2c and Figure S2) is comprehensively integrated within a compact footprint of 2.5 × 2.5 mm², accommodating 18 units that share a common ground end. Metal oxide-sensing nanomaterials are precisely loaded onto the underside of the chip by inkjet printing (Fig. 2d)^44,45^ and exposed to air after integration with CMOS readout circuits. Therefore, the microplate suspension membrane is strategically placed at the base of the etching trench, and it is shielded from any risk of breakdown. Under appropriate voltage conditions, the sensors exhibit distinct responses to varying types and concentrations of gases when operated at optimized temperatures ranging from 250 to 400 °C. Notably, our device demonstrated excellent heat-shielding performance, as illustrated in Fig. 2e. Although the temperature of the suspended membrane reached a peak of 367 °C, the corresponding infrared spectrum revealed minimal heat disturbance between the sensor units. This finding highlights the effectiveness of our device’s thermal management system in maintaining stable operating conditions.

To detect multiple gases, eight types of MOS nanomaterials are inkjet printed on the surface of microplates; these materials include SnO_2_, ZnO, Au-SnO_2_, WO_3_, Pt-SnO_2_, Fe_2_O_3_, TiO_2_, Pd-SnO_2_. Each is decorated on 2 sensor units. Sphere-like and polyhedron-like nanostructures with uniform sizes were clearly obtained on a large scale, as shown in Fig. 2f. The diameters of the SnO_2_, ZnO, Au-SnO_2_, WO_3_, Pt-SnO_2_, Fe_2_O_3_, TiO_2_, and Pd-SnO_2_ nanoparticles are 20 nm, 20–30 nm, 10–20 nm, 30–50 nm, 10–20 nm, 20–50 nm, 150–200 nm, and 10–20 nm, respectively. Compared to sensors based on nanowires and bulk crystals, sensors based on zero-dimensional MOS particles have good sensitivity^46^. Detailed information regarding MOS nanomaterials and their printing is provided in the Experimental Section and Table S2.

Benefiting from its exquisite design and precise manufacturing, this multifunctional MEMS sensor array exhibited excellent responses to gas, temperature and humidity (Fig. 2g–i). It took only 11 s for the response of 10 ppm acetone to reach the steady phase before reverting to the original state (Fig. 2g). The response is assessed by monitoring the relative resistance variation (ΔR/R_0_%) upon exposure, where R_0_ represents the initial resistance and ΔR signifies the resistance change after exposure to vapor. The sensing membrane is 150 × 150 μm² and is coated with SnO_2_ nanoparticles (the inset of Fig. 2g). Moreover, the olfactory sensing device exhibits remarkable long-term stability, with the heater resistance drifting by only approximately 3% after 100 days, as depicted in Figure S3. Figure S4 shows the simulated temperature of the microplates using the COMSOL Multiphysics Simulation package with a power consumption of 30 mW, revealing that the temperature of sensor 4 reaches 345 °C within 10 ms. These findings highlight the durability and rapid response capabilities of our sensing system. As shown in Figure S5, channel 1 was stable and reliable, with responses of 0.50 at 30 days and 0.48 at 60 days to 50 ppm acetone. Therefore, the sensor shows a slight change in response even after 2 months of testing, indicating good long-term stability and durability. Figure S6a and Figure S6b show the responses of the sensing array to 150 ppm CO under standard conditions (24 °C, 40% RH) and abnormal conditions (12 °C, 65% RH). Slight drifts of the channels are observed because of changes in humidity and temperature^47,48^.

In practical use, the influence of the environmental factors on the sensing system needs to be considered. The integration of temperature and humidity sensors is instrumental in mitigating the impact of environmental fluctuations on gas response, ensuring more accurate measurements. A platinum resistor, known for its temperature sensitivity, is employed to detect the surrounding environment’s temperature (Fig. 2h). A serpentine resistance wire with a width of 8 μm, is precisely fabricated on a 160 × 160 μm² suspended membrane (the inset of Fig. 2h). The corresponding temperature coefficient of resistance (TCR) reaches 0.00222/°C, exhibiting an approximately proportional relation between 8 °C and 100 °C; this result indicates its reliable response under ambient temperature variations. In addition, the moisture sensor (Fig. 2i), which features a 150 × 150 μm² membrane, is able to detect relative humidity levels ranging from 30–70% with a MoO_3_ coating^49^; this accurately reflects the environmental humidity information.

### Design and performance of the CMOS processing circuits

**Fig. 3:**
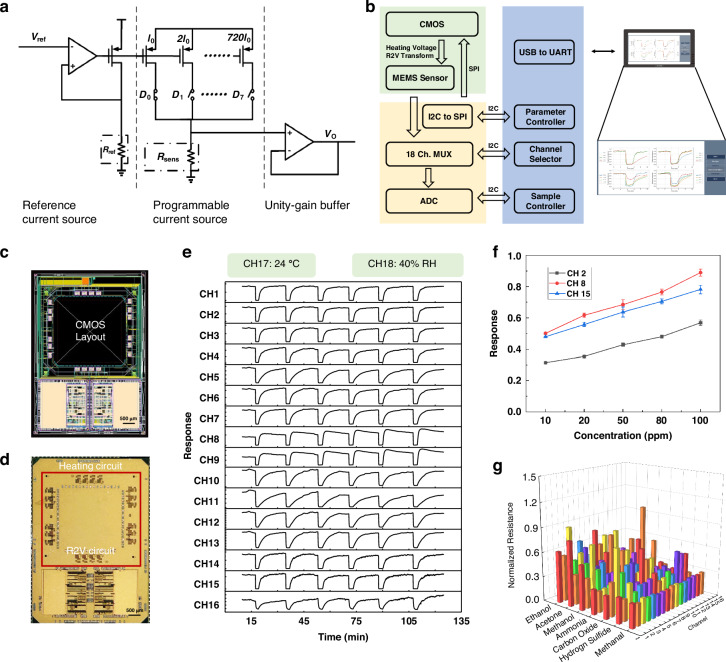
Design and performance of the CMOS circuit. **a** Design of the CMOS circuit structure. **b** Peripheral testing circuit structure. **c** CMOS circuit layout. **d** CMOS circuit chip, including a heating circuit and resistance-to-voltage circuit. **e** Multichannel cyclic testing results of the biomimetic intelligent olfactory sensing system. **f** Test results from Channels 2, 8, and 15 for the different concentrations of methanol. **g** Response results from the 16-channel gas sensors to different types of gases

To accurately record the sensing data of our device, the initial design of a CMOS readout circuit tailored for resistive gas sensors consists of a heating circuit and a resistance-to-voltage conversion circuit. The heating circuit is tasked with ensuring the normal operation of the sensor array by providing the necessary heat. The resistance-to-voltage conversion circuit facilitates the collection of gas responses at corresponding transmissions (Fig. 3a). It consists of a reference current source, a programmable current source, and a unity gain buffer. We further integrated the MEMS sensor array with the CMOS circuit through flip-chip bonding^50^. Vertical 3D integration between chips often entails intricate manufacturing procedures, commonly involving the incorporation of through-silicon vials (TSVs). In this study, we achieved vertical 3D integration with a readout circuit chip by optimizing the manufacturing process of inverting the MEMS chip. The layout design and resultant CMOS chip are depicted in Fig. 3c, d. Importantly, the entire CMOS-MEMS integration device spans a mere 5 × 8 mm^2^ with a thickness of less than 1 mm, demonstrating compactness without compromising functionality.

An extensive investigation into the gas sensing performance of our system was conducted. At a temperature of 25 °C and humidity of 40% RH, significant variations in responses were observed across the 16 channels toward 200 ppm ethanol in the cyclic test. This material demonstrated outstanding stability over six cycles (Figs. 3e and S9). Furthermore, the response of the biomimetic intelligent olfactory sensing system to varying concentrations of methanol (10, 20, 50, 80, and 100 ppm) was evaluated in 4–5 trials (Fig. 3f). The results indicated substantial disparities in responses among different channels to the same gas, while a linear trend in response to varying gas concentrations was evident within each channel. This finding highlights the system’s sensitivity and reliability in distinguishing diverse gases both in type and concentration. The comprehensive response of the 16-channel gas sensors to seven distinct types of gases (Table S1) is depicted in Fig. 3g, establishing a robust basis for their further application in gas identification.

### Gas recognition performance of the olfactory sensing system with the biomimetic lightweight olfactory machine learning strategy

**Fig. 4:**
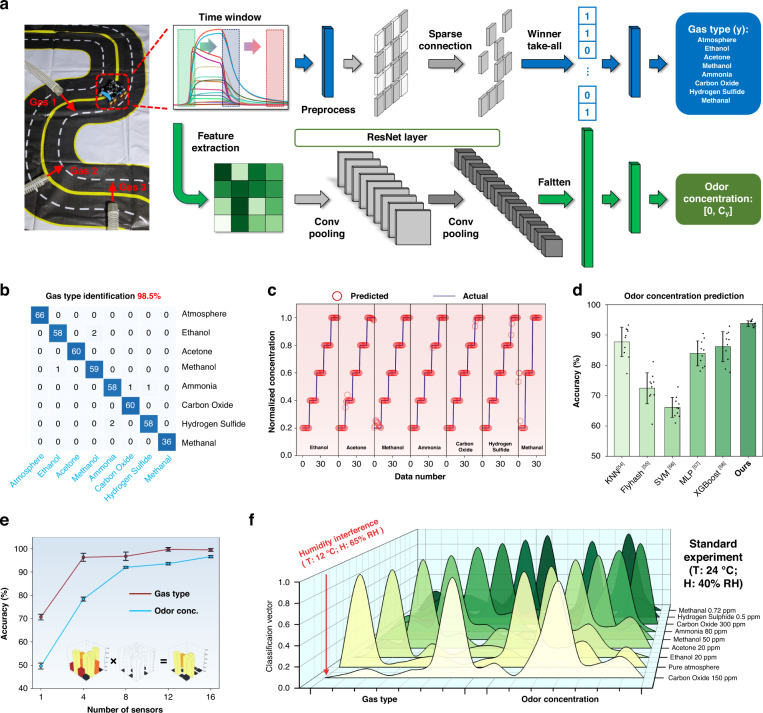
Biomimetic olfactory lightweight machine-learning strategy for gas type identification and odor concentration prediction. **a** Scheme of the neural network architecture used for gas type identification and odor concentration prediction from olfactory information in a simulated scenario. **b** Confusion matrix of the gas type identification strategy when recognizing eight environmental gases (including atmosphere, ethanol, acetone, methanol, ammonia, carbon oxide, hydrogen sulfide, and methanal). **c** Concentrations of the odor predicted by a biomimetic neural network algorithm. The data number refers to the number of data points used for algorithm processing. **d** Overall accuracy of the odor concentration prediction methods for comparison. The proposed network shows the best accuracy among the tested strategies. *n* = 10 for each group. The error bars denote standard deviations of the mean. **e** Prediction accuracy versus the number of sensors. The accuracy increases with more sensors. The illustration is a scheme showing damage to random parts of a gas sensor array. **f** Representative set of examples during the recognition of gas type and prediction of odor concentration in a practical environment, including a standard environment and humidity interference. The odor labels and corresponding classification vectors from the olfactory inputs are shown

Identifying the information of leaked gases in the environment is an important task of the system. The locality-sensitive hashing algorithm, inspired by the *Drosophila* neural circuit, was utilized for gas type identification and odor concentration prediction. Figure 4a illustrates the framework of the biomimetic olfactory machine learning strategy. Specifically, gas type is recognized through a shallow neural network inspired by *Drosophila*^51,52^ and is computed via a three-step procedure involving preprocessing, random sparse projection, and winner-take-all mechanisms. Subsequently, feature extraction and reconstruction are executed based on the olfactory information monitored within a defined time window. Moreover, the corresponding odor concentration is obtained by a modified version of the residual neural network^35^, and a detailed description can be found in the Experimental section. Since olfactory information is susceptible to environmental factors, we adopted a sparse connection operation to enhance the generalizability and robustness of the algorithm in challenging scenarios^37^^,53^.

The gas type identification strategy yields results displayed in the confusion matrix (Fig. 4b), indicating the high accuracy (98.5%) of our model on a testing dataset comprising 462 samples. Figure 4c shows the predicted concentrations using the sensing data from the first four experiments as the training dataset and the last sensing data as the testing dataset. Furthermore, the overall accuracy for comparing the proposed network with other methods is depicted in Fig. 4d. Compared to common algorithms such as kNN^54^, Flyhash^55^, SVM^56^, MLP^57^ and XGBoost^58^, our proposed network exhibits the highest odor concentration prediction accuracy (93.2%), demonstrating its potent online learning capability. Our algorithm is capable of delivering precise gas information in less than 100 ms.

Moreover, the olfactory sensing array likely experiences partial failure during practical application. Thus, we further designed and conducted experiments to assess recognition accuracy by intentionally disabling several sensors randomly. As shown in Fig. 4e, the accuracy of gas type identification and odor concentration prediction rapidly improves as the number of available sensors increases. The data become saturated after the number of sensors exceeds 8, aligning with the types of gas sensing materials utilized; these results exhibit the array’s adaptability under harsh conditions. The environmental temperature and humidity pose inevitable interference issues for gas sensors. Under standard conditions of 24 °C and 40% RH, the proposed algorithm can promptly and accurately recognize gas types and predict odor concentrations (Fig. 4f). Notably, the biomimetic olfactory system continued to operate effectively even under the influence of low temperature (12 °C) and high humidity (65% RH); these results show the system’s ability to resist disturbance and its robustness under various environmental conditions.

## Discussion and conclusion

Gas sensing systems have been in development for several decades, yet many of them consist of discrete sensor channels, board-level circuits and inefficient algorithms with a single function, bulky volumes, and low accuracy. In this study, we devised and manufactured an intelligent bionic olfactory sensing system integrated with a MEMS multichannel sensor array, a CMOS readout chip and *Drosophila*-inspired algorithms. Multifunctional environmental sensors (temperature and humidity sensors) eliminate surrounding interference in practical use. The flip-chip bonding process applied in vertical 3D integration reduces the manufacturing difficulty and overall volume. The combination of a shallow neural network and a residual neural network inspired by *Drosophila* ensures robust and accurate gas recognition both in quality and quantity.

Based on the exquisite design, optimized process, multifunctional integration, and bioinspired algorithm, our intelligent bionic olfactory sensing system is able to achieve gas type identification (7 types, 98.2%) and odor concentration prediction (33 concentrations, 93.2%), even in harsh scenarios involving damaged sensors (50% loss) and high humidity (65% RH). These results demonstrate its ability to recognize noxious gas under complex environmental conditions, thereby presenting opportunities for further application in medical care, environmental monitoring, and intelligent security alarm systems in emergency rescue.

## Experimental section

### Fabrication of the MEMS sensing array

Initially, a 200 nm layer of silicon oxide is thermally oxidized, followed by the utilization of reactive ion etching (RIE) to expose areas for patterning the interdigital electrodes and heating resistors. This silicon oxide layer serves to safeguard against undercutting during subsequent wet-etching processes. Subsequently, a 30 nm layer of adhesive Ta and a 300 nm layer of Pt film are deposited and patterned to form the interdigital electrodes for multichannel gas sensors. An insulated layer consisting of 200 nm of silicon oxide, 200 nm of silicon nitride, and 200 nm of silicon oxide was deposited using plasma-enhanced chemical vapor deposition (PECVD). Next, the heating circuit pads are exposed through RIE dry etching of the insulated layer. Another set of 30 nm adhesive Ta and 300 nm Pt film layers are deposited and patterned to create the heater elements. Silicon is deeply etched to a depth of 350 μm from the back of the wafer. Finally, the dielectric layer surrounding each sensor is dry etched, creating channels for anisotropic wet etching in a tetramethylammonium hydroxide (TMAH) solution to form suspension beam structures. In the last step, MOS nanomaterials are applied onto the interdigital electrode layer using inkjet printing.

### Inkjet printing

The 16 channels of the gas sensors were decorated with 8 different metal oxide-sensing nanomaterials, including SnO_2_, ZnO, WO_3_, Fe_2_O_3_, TiO_2_ (Onstar New Carbon Materials), Au-SnO_2_, Pt-SnO_2_, and Pd-SnO_2_ (Chenkona New Materials). Scanning electron microscopy images are shown in Fig. 2f. MOS nanomaterials are applied to an interdigital electrode using inkjet printing with the Standard Nanomaterial Deposition Inkjet Printing System Jetlab 4. The nanomaterials are dispersed with 5 wt% glycerol, 70 wt% ethylene glycol and 20 wt% dimethyl sulfoxide (DMSO) to form printing ink, several drops are printed on the surface of the microplates, and each drop is ~80 pL. Finally, the thicknesses of the metal oxide nanomaterial layers are ~5–10 μm.

### Design of the CMOS readout circuit

A heating circuit is employed to heat the sensor array, ensuring its normal operation. The resistance-to-voltage conversion circuit then transforms the resistance of the 18 sensors into voltage values. The heating circuit incorporates an adjustable output voltage linear regulator, providing a suitable temperature for the operation of gas sensors through an adjustable voltage. The circuit has a tunable output voltage linear regulator to furnish a suitable temperature for the gas sensors. The heating voltage can be precisely adjusted between 1.2 and 3 V using a register, with a voltage step of 0.1 V. Given the high temperature requirements during the operation of gas sensors, the resistance of the heater serving as the load is designed to be relatively small; thus, the load capacity of the linear regulator needs to be considered during the design phase. Here, every four MOS gas sensors share a linear regulator for temperature adjustment. The heating voltage can be freely adjusted between 1.2 and 3 V using a register, with a voltage step of 0.1 V. In addition, a bandgap reference circuit is needed to provide a voltage of 1 V as a reference voltage with a temperature drift characteristic not exceeding 15 ppm for the linear regulator. In Fig. 3a, the first segment is the reference current source, providing a 2 μA reference current for the subsequent programmable current source, where *R*_ref_ represents an external reference resistor. The second part is the programmable current source, which addresses the challenge of a limited output voltage swing (V_DD_ = 3.3 V) and substantial resistance variation of the sensor. A single current source alone may struggle to meet the requirements of a resistance-to-voltage conversion circuit. The third part is the unity gain buffer, which is needed to increase the load capacity of the programmable current source. To maximize the swing, the amplifier within the unity gain buffer needs to achieve rail-to-rail input and output capabilities^59,60^. Given the significant variation in the sensor resistance and the limited power supply voltage, different currents are used to ensure that overly large or small resistances do not cause the output voltage to exceed the power supply voltage range. Due to the substantial required output voltage variation, the output buffer needs to be designed for rail-to-rail operation.

### Integration of MEMS sensor array and CMOS circuit

During the layout design phase, in anticipation of the subsequent 3D integration of MEMS sensor arrays with CMOS circuit chips, the pad positions of both components were pre-aligned. Before integration, a layer of gold is sputtered onto the pads of the MEMS chip, followed by the deposition of gold balls with a diameter of ~50 μm (Figure S7). The height of the gold balls can compensate for any potential nonuniformities on the chip surface that may impede connectivity on one side. Subsequently, the flip-chip bonding with the CMOS chip is executed at a temperature of 340 °C, with a bonding duration of 50 s (Figure S8).

### Characterization of the electric olfactory sensing arrays

After the integration of CMOS and MEMS, further connections were established through wire bonding. The SPI port of the CMOS, along with the sampling ports of 18 channels of resistive sensors, was led out using wire bonding. These connections were made through pin headers to link with the sensor driver board. The pin headers not only facilitated the required power supply for the sensor board but also enabled the connection of the CMOS SPI port and the 18-channel resistive sensor sampling ports.

The CMOS chip primarily provided two sets of interfaces with MEMS sensors. First, a 16-channel programmable constant voltage source was used to configure the desired heating voltage on the 16 heating resistors of the MEMS sensors. Second, an 18-channel programmable current source was utilized to convert the resistance values from the 18 sensitive resistors on MEMS sensors into voltage values for ADC sampling on the sensor driver board. The current source could be programmed for different magnitudes of current output to adapt to varying resistance values across multiple orders of magnitude. In addition, the CMOS sensor driver chip included a set of serial peripheral interface (SPI) signal ports for receiving configuration information from the host computer, enabling programming of the current and voltage sources (Fig. 3b).

The sensor driver board consisted of several components and included a low-dropout linear regulator (LDO) power supply section based on the TPS79333 chip to provide power to the other sections on the driver board. An I2C-to-SPI protocol conversion circuit, based on the SC18IS602B chip, transformed the I2C protocol transmitted by the FPGA controller into the SPI protocol for driving the CMOS sensor driver chip. The combination of an ADC and an 18-channel multiplexer, based on the MAX1169 and ADG732 chips, respectively, was employed to collect voltage values obtained from the conversion of 18-channel sensitive resistors. An I2C protocol-to-GPIO (General Purpose Input & Output) circuit was used to drive GPIO ports through the GPIO protocol, further controlling the channel selection of the 18-channel multiplexer based on the TCA6416ARTWR chip. Communication between the FPGA control board and the sensor driver board utilized the I2C protocol, with a USB cable serving as its physical implementation, providing the necessary power supply for the sensor driver board. The FPGA controller, using Xilinx’s Spartan-6 chip as the control core, communicated with the host computer via a USB connection. This connection was established through a USB-to-serial chip on the FPGA development board, which was linked to the FPGA’s serial communication port.

### Gas test procedure

The gas testing system is placed within an 18 L glass chamber, and static gas blending is employed to test methanol, ethanol, and acetone. Dynamic gas blending is used for testing ammonia, hydrogen sulfide, carbon monoxide, and methanal gases. Five concentration gradients are selected for each gas (except for methanal, which is tested with three concentration gradients), and multiple cyclic tests are conducted for each concentration. A humidifier is utilized to create a low-temperature, high-humidity environment, and the resulting test data are employed for algorithmic processing.

### Dataset preparation

Seven gases were detected: ethanol, acetone, methanol, ammonia, carbon oxide, hydrogen sulfide, and methanal. We conducted 180 experiments in total. For each experiment, we selected 2 frames of data from the initial state as the atmosphere input and 12 frames of data from the stable state as the detected gas input. Therefore, our olfactory dataset included 2520 samples in total, and each consisted of 16 channel resistance values. To ensure the generalizability of the dataset, for every type and concentration in the experiments, we used a random set of data as the test set and the remaining data as the training set. There are a total of 2058 samples in the training set and 462 samples in the testing set.

Due to interference from operators and environmental factors, as well as accuracy issues with the collection equipment, the olfactory information needs to be denoised and normalized before inputting data into the model. The output value is first smoothed by a one-dimensional mean filter, and the resistance change ($\Delta R/{R}_{0}$) of the olfactory sensors is further processed by a normalization step. In terms of time window monitoring, for the olfactory signal sequence $\left({X}_{1},{X}_{2},\ldots ,{{X}}_{N}\right)$ of length *N*, ${X}_{{std}}$ represents the standard deviation of the olfactory signal within the time window. When ${X}_{{std}}$ exceeds the threshold, a sudden change in environmental gases is considered. The length of the time window and threshold can be adjusted according to the experimental environment.

### Gas type identification

The overall gas type identification scheme relies on the use of a *Drosophila* neural network^51^ to extract meaningful information from olfactory signals and classify gas types. The shallow neural network inspired by *Drosophila* first projects the 16-dimensional preprocessed input vector into a 640-dimensional hash space using a sparse, binary random matrix since a 40-fold expansion is present in the number of neurons from 50 projection neurons (PNs) to 2000 Kenyon cells (KCs) in the *Drosophila* neural circuit. Moreover, because each KC receives and sums the firing rates from approximately six randomly selected PNs, the sampling rate of the above random projection is approximately 10%. Finally, the proposed algorithm binarizes this representation by setting the indices of the top 32 elements to 1 and the remaining indices to 0 since the top 5% of Kenyon cells are retained, and the rest are silenced in the *Drosophila* neural circuit. As a result, we obtain an olfactory tag for gas type identification, which plays an important role in the entire algorithm.

### Odor concentration prediction

We used a modified version of the ResNet-18 architecture^35^ as the base of our network for odor concentration prediction. After completing gas type identification, we perform feature extraction and reconstruction to obtain the input of 4 × 4 pixels. Considering the input size and data characteristics, we remove the initial convolution layer, initial maximum pooling layer, and the upper two of the four ResNet layer groups, leaving the final feature vector size to be 128. The following two-layer fully connected neural network is used for the final odor concentration prediction. The dot product of the concentration weight and classification vector is the predicted concentration (Fig. S10).

Finally, we implemented the network in the PyTorch deep learning framework. We use the Adam solver implemented in PyTorch to train our model and minimize cross-entropy loss. We use a learning rate of 0.001. We train the network using our training dataset for 100 epochs with batches of 32 samples (results in Figure S11). We reported the average score of at least 10 training runs. Similar methods are also used for training and classification testing in other practical situations for evaluation.

### Numerical calculations

The numerical calculation is based on a commercial finite element method package (COMSOL Multiphysics 6.2). The heat transfer module and solid module are used to obtain the heat distribution of microplates under certain power consumption conditions. Heat conduction, convection and radiation are all considered.

## Supplementary information

Supplemental Material File #1
