# Supplementary material for: A Drosophila-inspired intelligent olfactory biomimetic sensing system for gas recognition in complex environments
Source: Microsyst Nanoeng. 2024 Oct 28;10:153. doi: 10.1038/s41378-024-00752-y (PMC11520895; doi:10.1038/s41378-024-00752-y)
Supplement: Supplementary file 1 — Supplemental Material File #1 [file 41378_2024_752_MOESM1_ESM.pdf]

Supporting Information

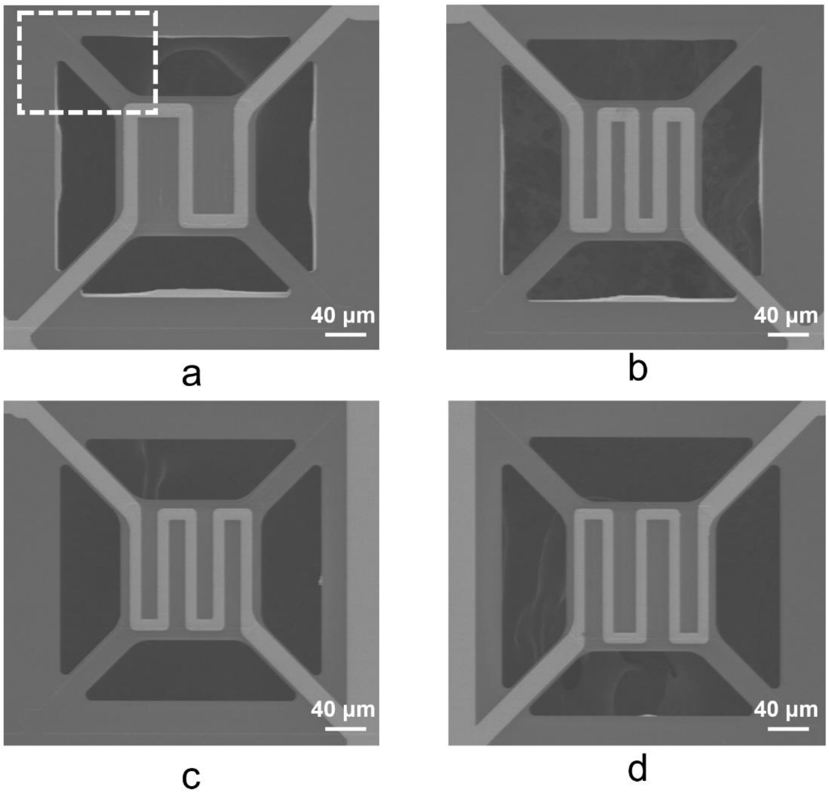

Figure S1: SEM image of the MEMS array. a. Sensor 1. b. Sensor 2. c. Sensor 3. d. Sensor 4.

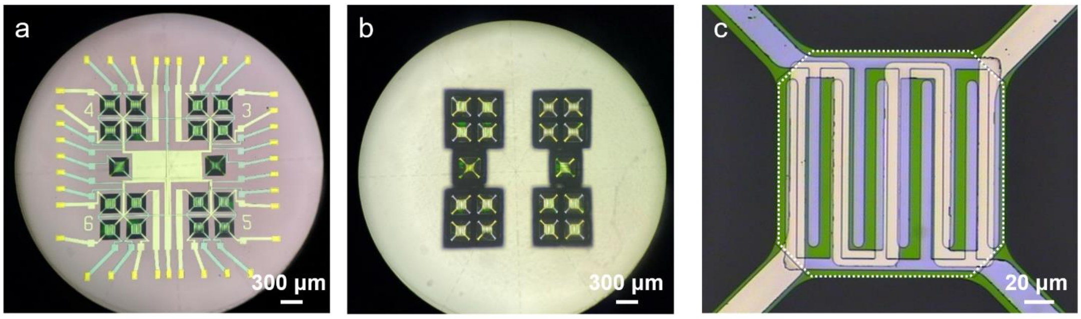

Figure S2: Upside(**a**) and downside(**b**) detail of the sensor chip. The total size is 2.5 × 2.5 mm^2. c. The suspension membrane of a gas sensor, the dotted area is exposed through dry etching to protect substrate from TMAH etching.

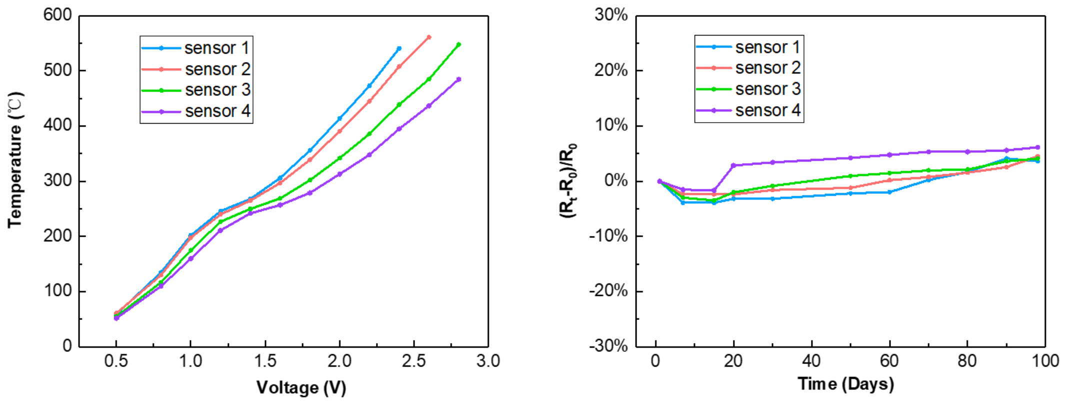

Figure S3: a. Temperature-Voltage relationship. b. Resistance variation of sensors in 100 days.

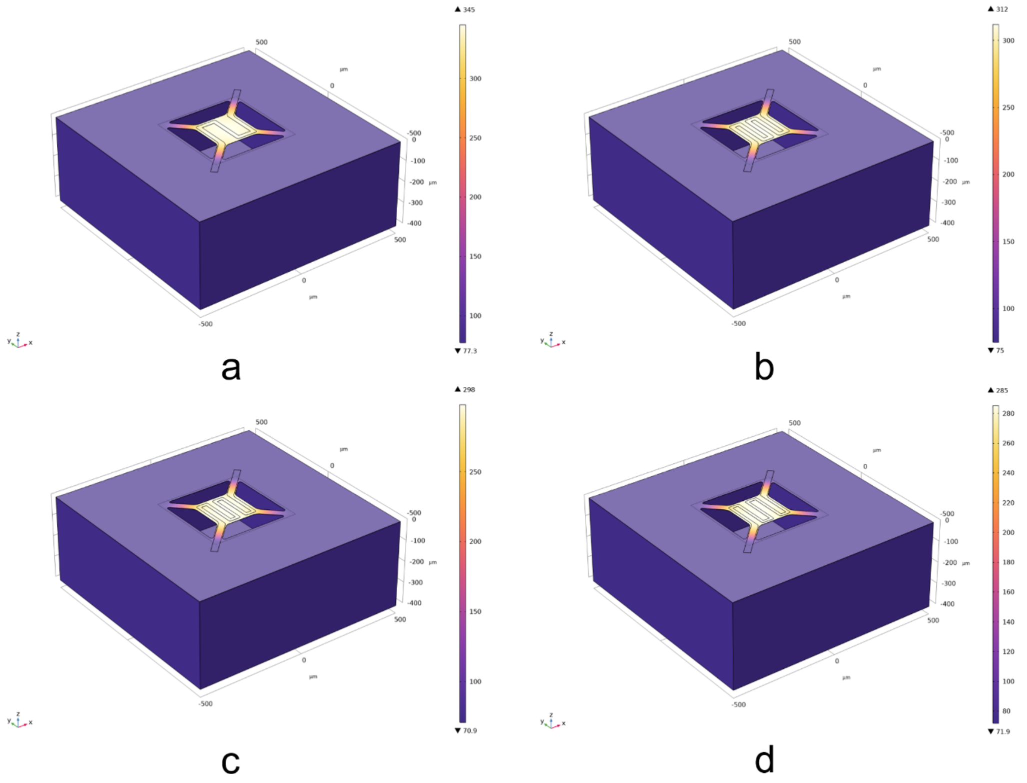

Figure S4: Temperature distribution under power consumption of 30mW in simulation experiment by COMSOL: a. Sensor 1; b. Sensor 2; c. Sensor 3; d. Sensor 4.

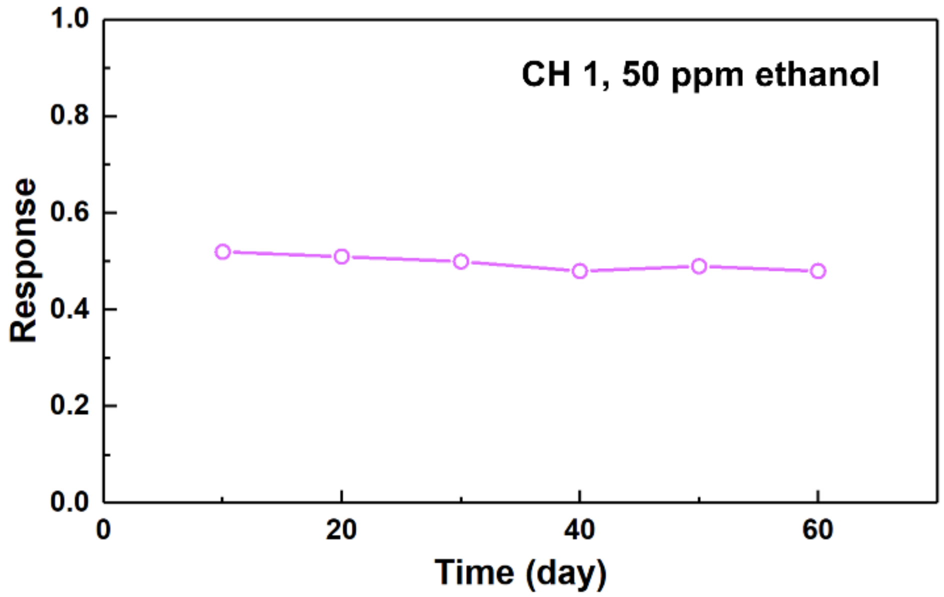

Figure S5: Response of channel 1 to 50 ppm acetone over 60 days.

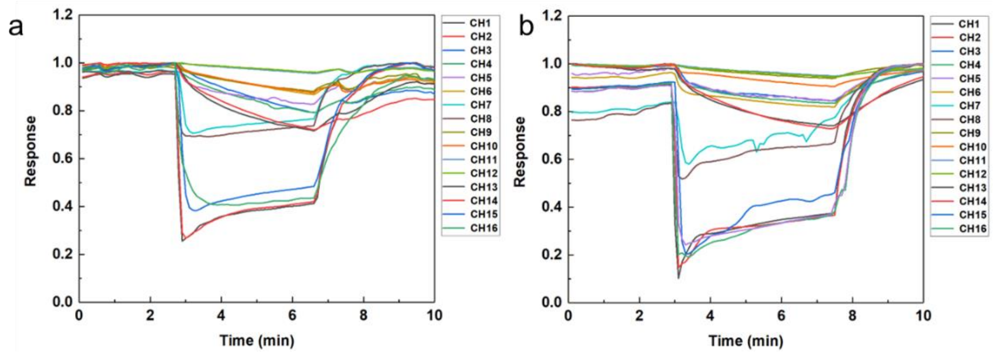

Figure S6: Response of sensing array of 150 ppm CO under standard circumstances (24℃, 40%RH) and abnormal environment (12℃, 65%RH)

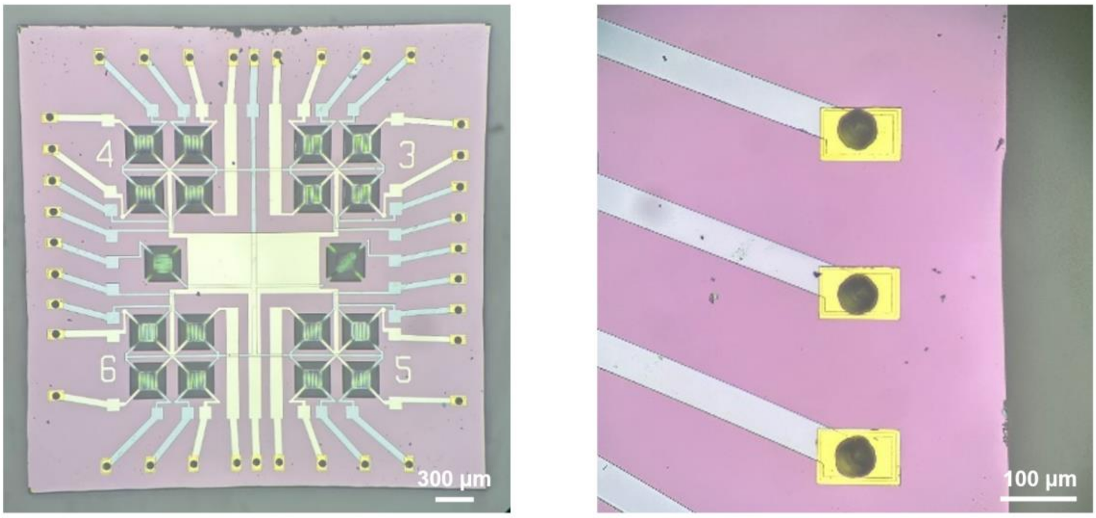

Figure S7: The deposition of gold balls on pads with a diameter of approximately 50 µm.

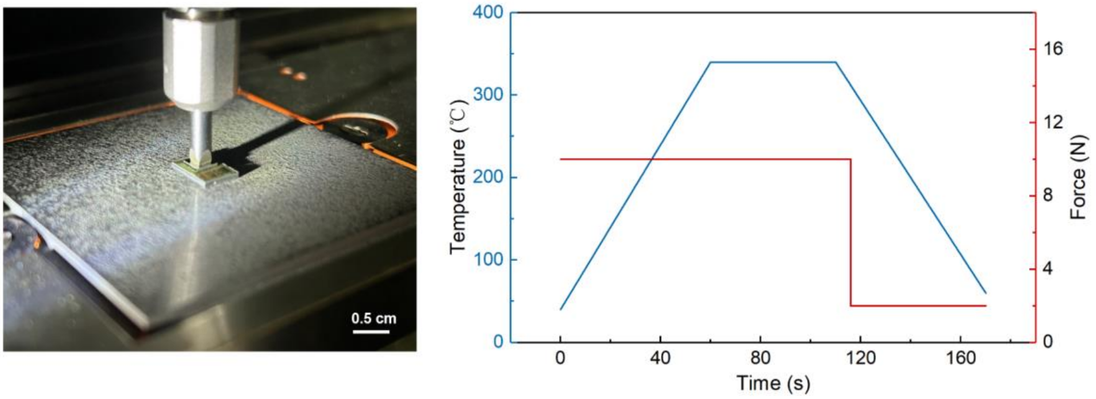

Figure S8: The flip-chip bonding with the CMOS chip is executed at a temperature of 340℃, with a bonding duration of 50 seconds.

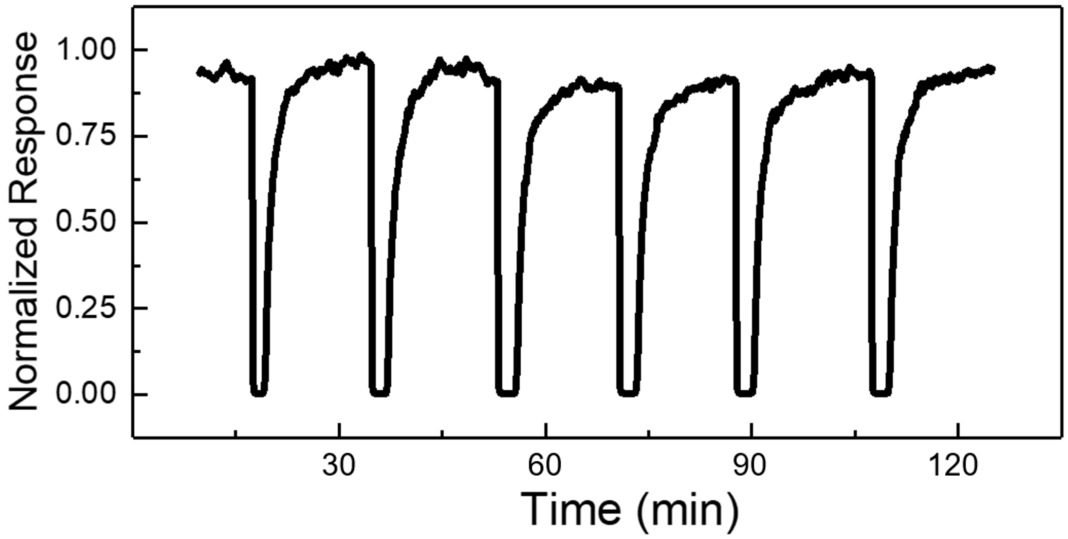

Figure S9: Detail response of channel-1 to 10 ppm methanal.

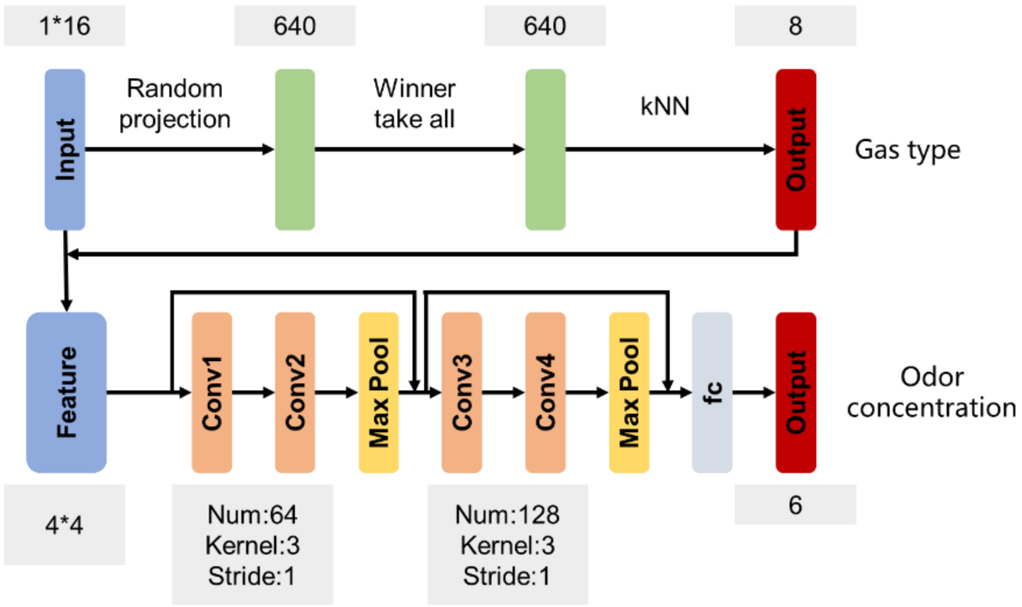

Figure S10: Architecture of biomimetic neural network algorithm for gas type identification and odor concentration prediction.

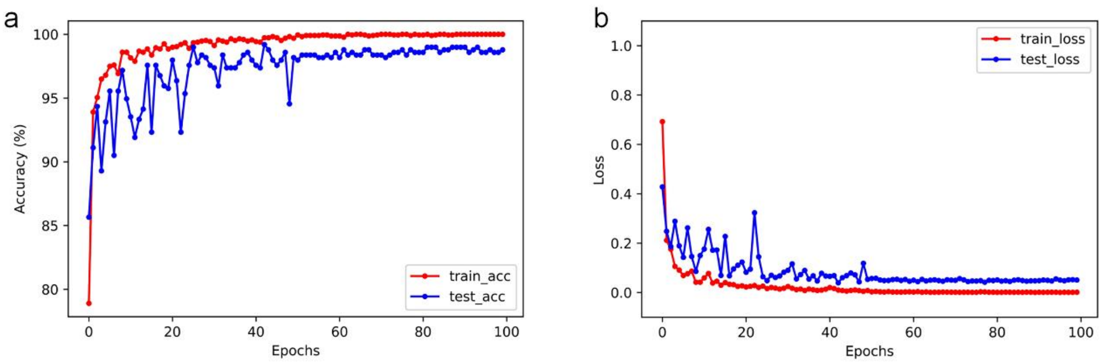

Figure S11: a. The change in recognition accuracy of the biomimetic neural network with the increase of training epochs. b. Loss of function variation during the training process.

Table S1: Gas test of different types and concentrations of gases.

Gas Type	Concentration（ppm)				
Carbon Oxide	100	150	200	250	300
Ammonia	20	40	60	80	100
Methanol	10	20	50	80	100
Acetone	10	20	50	80	100
Ethanol	20	50	100	150	200
Hydrogen Sulfide	0.2	0.5	0.8	1	1.5
Methanal	0.18		0.36		0.72

Table S2: Information of MOS nanomaterials.

**MOS materials**	**Diameter (nm)**	**Dopant concentration**	**Channel**	**Printing ink**
SnO_2	20	/	1,2	5wt% glycerol, 70wt% ethylene glycol and 20wt% DMSO
ZnO	20-30	/	3,4	
Au-SnO_2	10-20	1.5wt%	5,6	
WO_3	30-50	/	7,8	
Pt-SnO_2	10-20	1.5wt%	9,10	
Fe_2O_3	20-50	/	11,12	
TiO_2	150-200	/	13,14	
Pd-SnO_2	10-20	1.5wt%	15,16	
